# Repositioning of Antiparasitic Drugs for Tumor Treatment

**DOI:** 10.3389/fonc.2021.670804

**Published:** 2021-04-29

**Authors:** Yan-Qi Li, Zhi Zheng, Quan-Xing Liu, Xiao Lu, Dong Zhou, Jiao Zhang, Hong Zheng, Ji-Gang Dai

**Affiliations:** Department of Thoracic Surgery, Xinqiao Hospital, Army Medical University (Third Military Medical University), Chongqing, China

**Keywords:** drug repositioning, antiparasitic drugs, macrolides, benzimidazoles, artemisinin, quinolines, autophagy, ferroptosis

## Abstract

Drug repositioning is a strategy for identifying new antitumor drugs; this strategy allows existing and approved clinical drugs to be innovatively repurposed to treat tumors. Based on the similarities between parasitic diseases and cancer, recent studies aimed to investigate the efficacy of existing antiparasitic drugs in cancer. In this review, we selected two antihelminthic drugs (macrolides and benzimidazoles) and two antiprotozoal drugs (artemisinin and its derivatives, and quinolines) and summarized the research progresses made to date on the role of these drugs in cancer. Overall, these drugs regulate tumor growth *via* multiple targets, pathways, and modes of action. These antiparasitic drugs are good candidates for comprehensive, in-depth analyses of tumor occurrence and development. In-depth studies may improve the current tumor diagnoses and treatment regimens. However, for clinical application, current investigations are still insufficient, warranting more comprehensive analyses.

## Introduction

There exists a close connection between parasitic infections and cancer (1–3). Helminth infections are widespread the world over, and the causative parasites are thought to be responsible for causing cancer in humans (4). Thus far, *Schistosoma haematobium*, *Clonorchis sinensis*, and *Opisthorchis viverrini* have been recognized as clear biological carcinogens (1). The specific carcinogenic mechanism is not yet clear, and the metabolites of catechol estrogens and parasite-derived oxysterols may play an important role (5). Unlike worms, protozoa have not been identified as biological carcinogens; however, certain characteristics of protozoa are similar to those of cancer. In a manner similar to the immune evasion strategies employed by cancers, *Trypanosoma cruzi* and Leishmania parasites leverage the immune mechanisms to persist in the body and establish a chronic infection (6). Although malaria is the most widespread parasitic disease in the world, it does not seem to be carcinogenic (2). However, the incidence of malaria is positively correlated with mortality in most cancers, with the exception of colorectal, lung, gastric, and several other types of cancer whose mortalities exhibit an inverse correlation with malaria (7, 8). Thus, the relationship between malaria and cancer is worth exploring.

Cancer is the second leading cause of death worldwide and is a major burden of disease (9, 10). However, with proper treatment, many cancers can be cured. Drugs are essential in the treatment of tumors but are often not as effective as required because of drug resistance and low specificity (11). Despite the emergence of highly specific monoclonal antibody drugs, the drugs are unsuitable for a wide range of clinical applications because they have strict requirements for the target (12). Therefore, the development of new antitumor drugs is still urgently needed. However, owing to the similarities between parasitic diseases and cancer as well as the successful clinical administration of antiparasitic drugs for years, it seems feasible to repurpose existing antiparasitic drugs into antitumor drugs. In fact, some antiparasitic and antitumor drugs share the same target, a variety of drugs targeting CDKs, TGR enzyme, tubulin/microtubule system have been confirmed to have dual effects on anti-parasites and anti-cancer (13).

Research on the repurposing antiparasitic drugs for tumors has gradually gained popularity. However, many studies have reported contradictory results. We selected two well-researched antihelminthic drugs and two antiprotozoal drugs and summarized the corresponding research progress to provide direction for further exploration into the repurposing of antiparasitic drugs as antitumor drugs.

## Progress on the Use of Macrolide Antiparasitic Drugs for Treating Cancer

**Figure 1:**
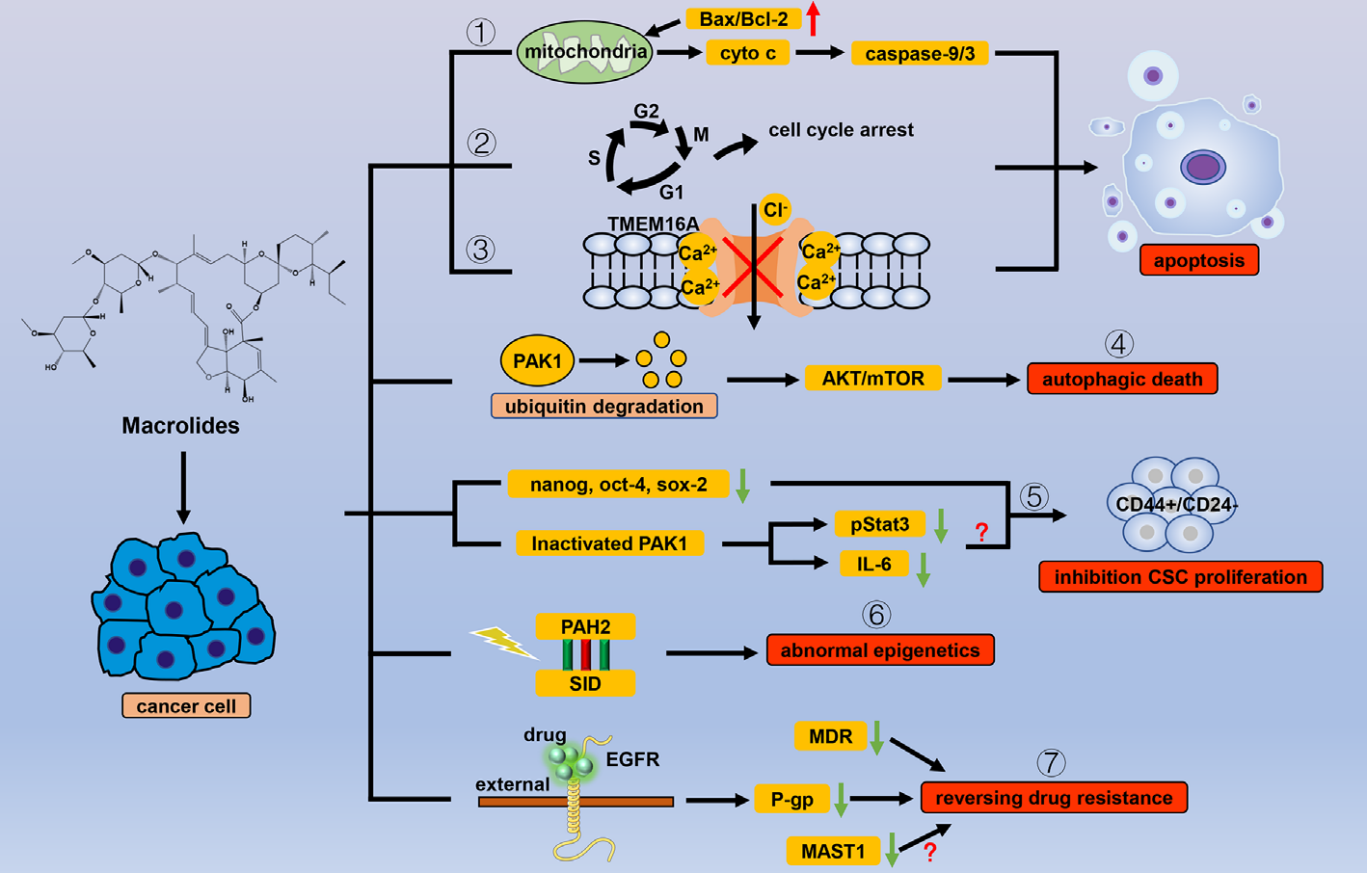
Efficacy of macrolide antiparasitic drugs in cancer. Apoptosis is the chief mechanism used by macrolide drugs to kill cancer cells. Macrolides trigger apoptosis through the (1) mitochondrial pathway, (2) cell cycle arrest, and (3) inhibition of the current of Ca^2+^ ion-activated Cl^-^ channels. Other than apoptosis, macrolides can cause autophagic death of cancer cells by (4) degrading PAK1. When used for cancer cells, macrolides show selectivity for CSCs by (5) inhibiting stem cell genes and inactivating PAK1. Macrolides also reverse the abnormal epigenetics of tumor cells through (6) the combination of PAH2 and SID domain. By binding to the extracellular segment of EGFR, macrolides can (7) inhibit the transcription of P-gp, thereby reversing tumor resistance in which MDR and MASTA1 are also involved.

Macrolides are antiparasitic drugs with dual functions *in vitro* and *in vivo*. The mechanism of macrolides is mainly to increase the concentration of the inhibitory transmitter GABA and enhance the permeability of the nerve membrane to chloride ions, causing neuromuscular paralysis and death (14). Macrolides have long been used to kill nematodes and consist of two main categories: avermectins and milbemycins. Apart from the antiparasitic effects, macrolide drugs also show different levels of anticancer activity (**Figure 1**).

### Avermectins

Commonly used avermectins include avermectin (AVM), ivermectin (IVM), doramectin (DRM), eprinomectin (EPM), and selamectin (SLM). Although all these avermectins show anticancer activity, studies on IVM are more comprehensive than those on other drugs. IVM can regulate the natural progression of tumors *via* multiple pathways.

Apoptosis is an important mechanism used by IVMs to kill cancer cells. Eukaryotic translation initiation factor 4A isoform 3 (EIF4A3) is an RNA-binding protein involved in the splicing modulation of BCL2L1/Bcl-X and is considered to be closely associated with apoptosis. SILAC-based quantitative proteomic analysis revealed that IVM inhibited the expression of EIF4A3 and 116 EIF4A3-binding mRNAs (15). In LA795 cells, IVM analogs (IVM, EPM, and SLM) can significantly inhibit currents mediated by the transmembrane member 16A (TMEM16A), an endogenous Ca^2+^-activated Cl^-^ channel closely related to tumorigenesis, thereby inducing apoptosis (16). In addition, IVMs utilize the well-studied pathway of mitochondrial apoptosis to exert their anticancer activity. When acting on HeLa cells, IVM can increase the ratio of Bax/Bcl-2 and induce release of mitochondrial cytochrome c into the cytoplasm, thus stimulating caspase-9/-3-mediated apoptosis (17). In chronic myeloid leukemia and renal cell carcinoma cells, IVM can induce apoptosis by inducing mitochondrial dysfunction (18).

Cell cycle arrest can easily lead to apoptosis. Several studies have revealed that in cancer cells, IVM arrests the cell cycle in different phases by regulating the expression of proteins that control the cell cycle, thereby inducing apoptosis (19–22). Especially in epithelial ovarian cancer, IVM induces apoptosis through multiphasic cell cycle arrest, and exhibits KPNB1-dependent antitumor effects (22). The cell cycle is also the target of many chemotherapeutic drugs, and combinatorial treatment with IVM and clinical drugs is worth investigating. In fact, in multiple *in vivo* and *in vitro* experiments, IVM significantly enhanced the efficacy of various drugs, including cisplatin and tamoxifen, but this result still needs clinical verification (20, 23).

Autophagy regulation is another important mechanism underlying IVM action. A study on breast cancer revealed that when IVM was applied to cancer cells, no obvious apoptosis was observed before 24 h of treatment, but the inhibition of growth of these cancer cells during the 24 h was evident. Later in that study, autophagy flux increased during the first 24 h of IVM treatment, and the anticancer effect during this period was reversed when IVM was used to treat cancer cells Beclin 1 or Atg5 knockdown (24). Current studies argue that IVM degrades PAK1 in cancer cells through the ubiquitination pathway, thereby inactivating the AKT-mTOR pathway, which is the key negative regulatory pathway of autophagy (24, 25). Although mechanisms used by IVM require more detailed exploration, it is encouraging that more studies indicate that induction of autophagy may be used as a method of synergistic treatment in clinical tumor chemotherapy, highlighting the potential of IVM as a clinical adjuvant drug (26, 27).

In addition to the anticancer mechanism, the selective functional characteristics of IVMs are also noteworthy. IVMs exhibit more pronounced toxicity toward cancer cells than toward non-cancer cells, which may be related to higher mitochondrial biogenesis in cancer cells (28). More importantly, when acting on cancer cells, IVM still exhibits different levels of cytotoxicity in different cancer cell subgroups. The CD44+/CD24- subpopulation of breast cancer cells have been previously reported to possess stem/progenitor cell properties (29, 30). IVM preferentially inhibits the viability of CD44 +/CD24- subpopulation cells (cancer stem cells (CSCs)) and reduces the expression of stemness genes (NANOG, POU5F1, and SOX2) (31). Current research points out that this may be related to the ability of IVMs to inactivate p21-activated kinase (PAK1), thereby reducing the levels of pStat3 and extracellular IL-6 and inhibiting the formation of CSCs (32). Research on the specific effect of IVM on CSCs is still limited, and many other knowledge gaps exist that require further research. In general, the selective nature of IVMs shows that it is almost non-toxic to non-cancer cells but can effectively inhibit the growth of cancer cells, demonstrating its unique potential as an anticancer drug.

IVMs have also shown anticancer capabilities in many fields other than these mentioned above. Although research on these aspects is rare, it has broadened the scope of exploration of IVMs. The latest research showed that IVMs could reverse tumor resistance. IVM at a low dose that does not produce evident cytotoxicity can bind to the extracellular domain of EGFR, which inhibits the activation of EGFR and its downstream signaling cascade ERK/Akt/NF-κB, thus inhibiting the transcription factor NF-κB and leading to reduction in P-glycoprotein (P-gp) transcription (33). Moreover, in triple-negative breast cancer (TNBC), the targeted disruption of the Sin3 (a master transcriptional scaffold and corepressor that plays an essential role in the regulation of gene transcription and maintenance of chromatin structure) complex by introducing a Sin3 interaction domain (SID) decoy that interferes with PAH2 binding by sequestering SID-containing partner proteins reverted the silencing of genes involved in cell growth and differentiation (34–36). Interestingly, IVM and SLM can be used as small molecule inhibitors of SID peptides that play a similar role to that of Sin3 disruption, indicating that AVMs can also exert anticancer effects by regulating the abnormal epigenetics of tumors (37). Furthermore, the activation of WNT-TCF signaling is implicated in multiple diseases, but there are no WNT-TCF antagonists in clinical use. However, SLM and IVM can reduce the expression of target proteins in this pathway by mimicking dnTCF, further demonstrating the application potential of these drugs (38).

### Milbemycins

The milbemycin family comprises a series of 16-membered macrolide antibiotics that contain a highly characteristic spiroketal group that can be produced by several Streptomyces, these antibiotics have strong biological activities and are used as highly selective and potent broad-spectrum antiparasitic agents (39–41). At present, research on milbemycins in cancer was relatively rare, and milbemycins have been found to play an important role in reversing tumor drug resistance. Milbemycins can restore the sensitivity of cancer cells toward chemotherapy drugs by reducing the expression of MDR1 or P-gp, and its concentration has no obvious cytotoxic effect on cancer cells (42, 43). Cisplatin (DDP) is one of the most widely used chemotherapeutic drugs and is considered the first-line treatment for many cancers, but drug resistance limits its therapeutic potential. A recent study found that serine/threonine kinase 1 (MAST1) was a major driver of DDP resistance in human cancers (44). Encouragingly, in multidrug and cisplatin-resistant human lung adenocarcinoma (A549/DDP) cells, a milbemycin compound isolated from *Streptomyces* sp. FJS31-2, named VM48130, reduced the expression of multiple resistance-related genes, including MAST1, to reverse resistance, which further demonstrated the potential of milbemycin as an adjuvant drug in clinical chemotherapy (45). The anticancer mechanisms of these compounds also include other aspects. For example, moxidectin effectively inhibited the proliferation of rat C6 and human U251 glioma cells. Mitochondria-related apoptotic pathways, cell cycle arrest, and autophagy induced by the AKT/mTOR signaling pathway in cancer cells are all considered to be involved in this process, but the specific mechanism remains to be explored (46, 47).

## Progress on the Use of Benzimidazole Antiparasitic Drugs for Treating Cancer

**Figure 2:**
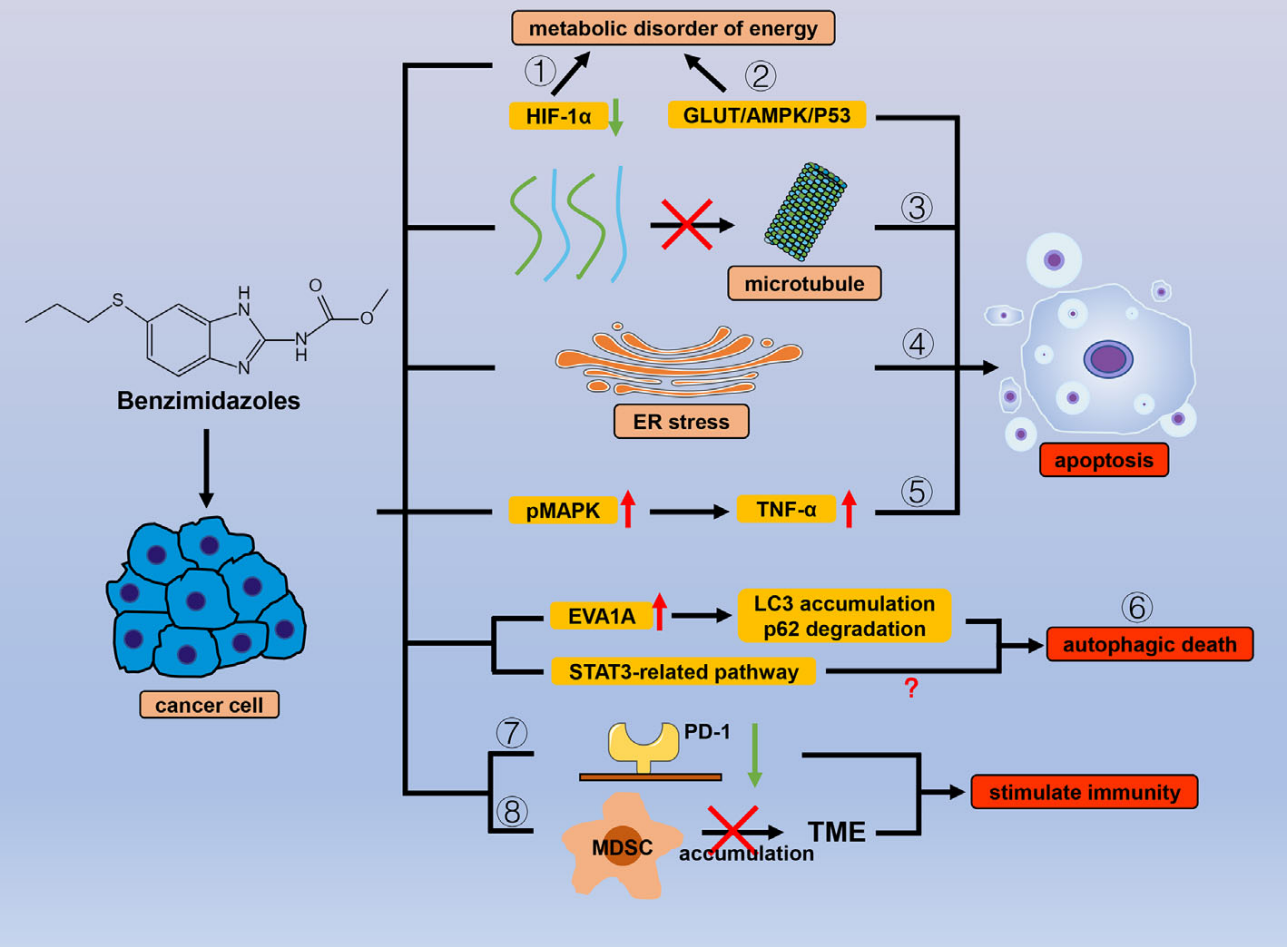
Efficacy of benzimidazole antiparasitic drugs in cancer. Benzimidazoles can cause energy metabolism disorders and reduce cancer cell tolerance to hypoxic environments by (1) inhibiting the expression of HIF-1α and (2) inhibiting sugar intake through the GLUT/AMPK/P53 pathway. Apart from sugar metabolism, benzimidazoles also induce apoptosis by (3) inhibiting microtubule polymerization and (4) ER stress, and (5) promoting MAPK phosphorylation. By affecting numerous autophagy-related proteins (LC3, P62, and EVA1A) and downstream signals related to STAT3, benzimidazole can also cause (6) autophagic cancer cell death. (7) Benzimidazoles reduce the expression of PD-1 and (8) inhibit the accumulation of MDSC in the TME to stimulate antitumor immunity.

Benzimidazole is a broad-spectrum antiparasitic drug with a structure similar to that of purines and is mainly used in clinics for nematodes. Benzimidazoles include albendazole (ABZ), flubendazole (FLU), fenbendazole (FBZ), oxibendazole (OBZ), and febantel (FBT). In general, these drugs mainly exert their antiparasitic effects by interfering with sugar metabolism, affecting adenosine triphosphate (ATP) production, and binding to tubulin to affect the cell cycle (48, 49). These biological processes are also critical in cancer, and it seems inevitable that such drugs are effective in tumor treatment. Correspondingly, many studies have shown that benzimidazoles have prominent anticancer activity (**Figure 2**).

### Albendazole

Based on the antiparasitic mechanism, studies have found that ABZ can inhibit glucose uptake through the GLUT1/AMPK/P53 signaling pathway, thereby disrupting sugar metabolism in cancer cells and inducing cell apoptosis (50). ABZ, a microtubule-targeting agent (MTA), induces apoptosis by disrupting microtubule formation and causes mitotic arrest in tumors (51, 52). ABZ causes bundles of short microtubules to form along the edges of cells rather than covering the entire cell, leading to a series of biological reactions (52). MTAs are a class of drugs currently used in chemotherapy. A synergistic antiproliferative effect is observed upon using combinatorial therapy involving low concentrations of ABZ, colchicine, and ABZ plus 2-methoxyestradiol (2ME) (53). However, recent studies have revealed an interesting mechanism underlying the anticancer activity of ABZs, i.e., targeting the microtubules. In K562 cells, when compared with paclitaxel and other MTAs, ABZ treatment significantly increased the number of cells arrested at the G2/M phase in a short time, and ABZs did not immediately activate apoptosis. Subsequently, ABZs could upregulate SIRT3 expression, which is believed to regulate SOD2 activity to clear mitochondrial reactive oxygen species (ROS). They further speculated that this ability allowed ABZs to protect cancer cells from cytotoxicity in the short-term, but when SIRT3 expression was further reduced, this unique ABZ mechanism was no longer effective (54, 55).

Whether it be inhibiting the glucose metabolism pathway or targeting microtubules to exert anticancer activity, triggering cancer cell apoptosis is the common end result. In fact, ABZs can induce apoptosis in other ways. According to a study on cutaneous squamous cell carcinoma, ABZs increase apoptosis-related signals by inducing endoplasmic reticulum (ER) stress, and pretreatment with the ER stress inhibitor 4-PBA attenuates ABZ-induced apoptosis (56). Furthermore, in human leukemia U937 cells, ABZs increase MAPK phosphorylation and upregulate TNF-α expression, thus inducing apoptosis. The same pathway is seemingly involved in the ABZ-induced death of HL-60 cells (57).

Moreover, most cancer cells generate ATP using accelerated glycolysis rates, and glucose is converted to lactate instead of being metabolized by oxidative phosphorylation, even when oxygen is abundant (58, 59). Current research indicates that hypoxia-inducible factor-1α (HIF-1α) plays a critical role in this process. In general, under hypoxic conditions, HIF-1α maintains the survival requirements of cancer cells by regulating the expression of a series of glycolytic enzymes and can also bind to the vascular endothelial cell growth factor (VEGF) gene promoter to induce VEGF expression and angiogenesis (60, 61). Therefore, HIF-1α is becoming an increasingly attractive therapeutic target in the treatment of cancer. It is encouraging that ABZs significantly inhibited the expression of HIF-1α in non-small cell lung cancer and ovarian cancer; however, ABZ treatment did not affect the HIF-1α mRNA level, suggesting that other unknown regulatory pathways may be involved in this process (62, 63).

Research institutions have already carried out phase I clinical trials of oral ABZ to treat advanced cancer patients to detect its maximum tolerated dose. Results from the 36 patients with refractory solid tumors enrolled in the study showed that the recommended dose for further study was 1,200 mg twice daily for 14 days in a 21-day cycle, with myelosuppression being the main dose-limiting toxicity (64). Although no patients achieved partial or complete response according to the RECIST study’s criteria, 4 out of 24 patients with assessable tumor markers (16%) demonstrated a decrease in tumor markers of more than 50%. In contrast, another patient had a significant decrease in tumor markers and a prolonged period of stable disease. Overall, as research continues to progress, new anticancer drugs based on ABZ can be expected.

### Flubendazole

Similar to ABZ, FLU induces monopolar spindle formation by inhibiting tubulin polymerization, inhibiting proliferation and migration, ultimately triggering apoptosis in a variety of cancer cell lines (65–69). However, more distinctive is that autophagy seems to play an important role in the anticancer activity of FLU. Using molecular docking simulation technology to screen numerous small molecule drugs approved by the Food and Drug Administration (FDA), FLU was found to have the highest antitumor activity and the ability to target autophagy-related gene (ATG) 4 B. Molecular dynamics simulation revealed that FLU bound with high affinity to ATG4B protein, and that it could induce autophagy and exhibit an antiproliferative effect on TNBC cancer cells (70). The latest research, however, has proposed another possible mechanism for FLU’s anticancer activity on TNBC cells. FLU treatment promotes autophagy by upregulating the expression of Eva-1 homolog A (EVA1A), a protein involved in autophagy and apoptosis-induced cell death (71). EVA1A knockdown, results in the partial inhibition of LC3 puncta accumulation, p62 degradation, and LC3 lipidation in TNBC cells (72). FLU also promotes autophagy in other malignant cell lines, such as A549 and H460 (73), through the regulation of the signal transducer and activator of transcription 3 (STAT3)-related pathway to induce apoptosis in human colorectal cancer cells, but the specific mechanism remains unclear (74).

Moreover, FLU has clinical value because it is potentially involved in tumor immunotherapy and molecular targeted drug resistance. Programmed cell death protein-1 (PD-1) and programmed cell death-ligand 1 (PD-L1) are immune system regulators that play a role in dampening the immune response to cancer cells, and PD-1 inhibitors have already changed the paradigm of cancer treatment in many cancers (75, 76). However, all available PD-1/PD-L1 treatments are antibodies that require intravenous infusion, resulting in exorbitant costs. PD-1/PD-L1 treatments can also have unpredictable and/or poor response in second-line treatment; therefore, finding a small molecule inhibitor is more convenient (77). A study on melanoma showed that FLU could inhibit the expression of PD-1 in cancer cells and the accumulation of myeloid-derived suppressor cells (MDSCs) in the tumor microenvironment, indicating its ability to elicit the host’s antitumor immunity, but the specific mechanism remains to be explored (78). In general, this suggests huge potential application of FLU in tumor immunotherapy. Apart from this, trastuzumab provides significant clinical benefit for HER2-positive breast cancers, but nearly 70% of patients experience primary or acquired resistance, which dramatically limits the therapeutic effect (79). Encouragingly, FLU significantly reduced p95HER2 expression and the phosphorylation level of HER2, HER3, and AKT, preventing the hetero-dimerization of HER2/HER3 in trastuzumab-resistant cells (80), which play an important role in trastuzumab resistance (81–83). Combination therapy with FLU seems to be a possible solution to improve the efficacy of trastuzumab.

## Progress on the Use of Artemisinin and Its Derivatives for Treating Cancer

**Figure 3:**
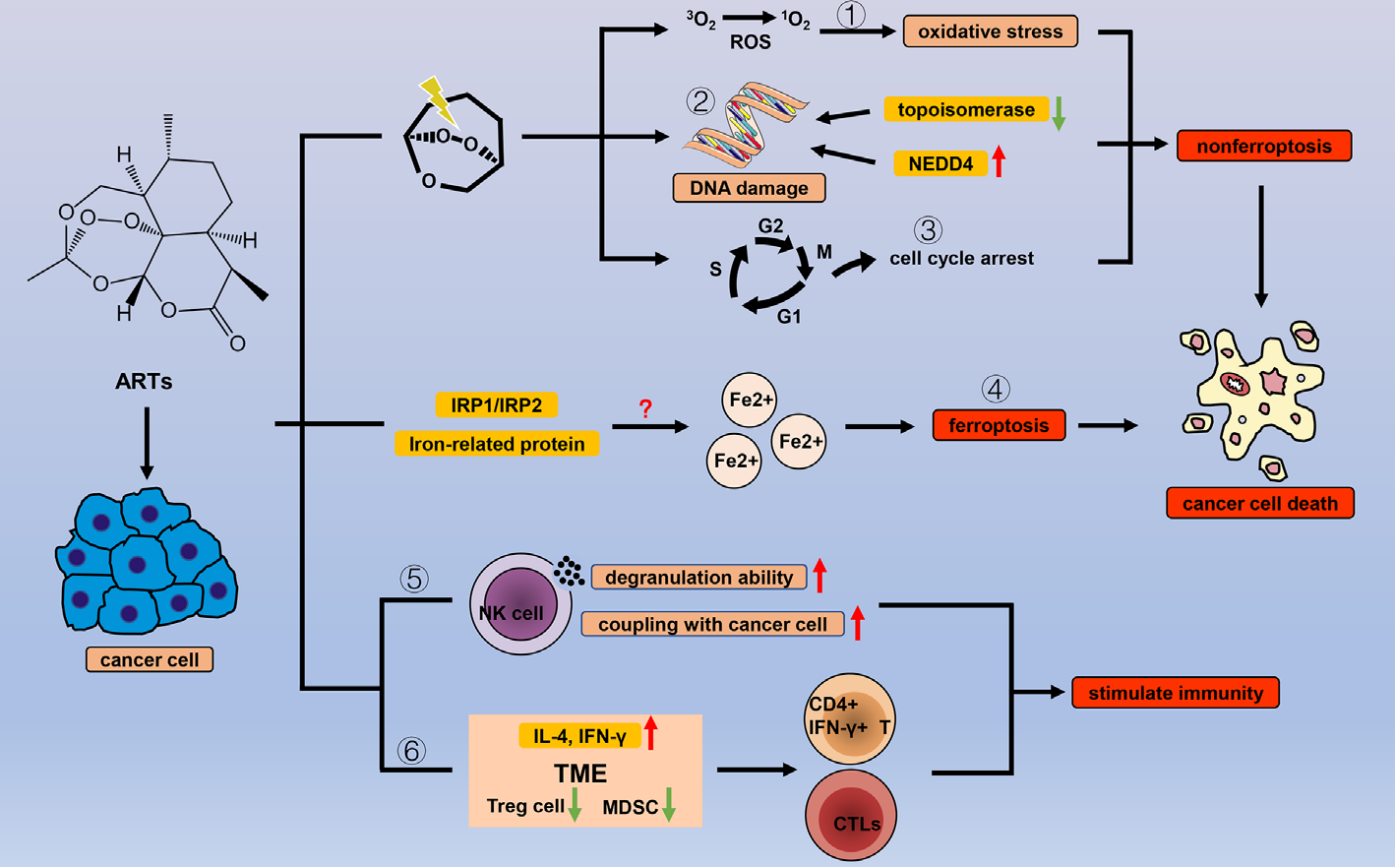
Efficacy of ARTs in cancer. With the help of endogenous peroxides, ARTs can cause oxidative stress by (1) increasing the concentration of ROS, (2) DNA damage, and (3) cell cycle arrest to cause cancer cell death. In addition to the common types of cell death, ARTs increase the concentration of unstable iron ions in cancer cells by (4) regulating a variety of iron-related proteins and IRP1/IRP2, thereby triggering ferroptosis. ARTs strengthen the cancer-killing effect of NK cells by (5) enhancing their degranulation ability and increasing the connection between NK cells and cancer cells. ARTs (6) reduce negative regulation factors (Treg cells and MDSCs) and increase IL-4 and IFN-γ in TME to stimulate T cell immune response.

Artemisinin (ARS) is a 1,2,-trioxane from the Chinese medicinal plant Sweet Wormwood, and since its antimalarial effect was discovered, research on ARS has been continuously focused on. A variety of ARS and its derivatives (ARTs), including dihydroartemisinin (DHA), artemether (ARM), artesunate (ART), and artemisitene (ATT), have emerged because of the advancements of drug modification and synthesis technology. ARS-based combination therapies are established standard treatments for malaria worldwide (84–87). It is currently considered that the heme-irons released by *Plasmodium*-attacking red blood cells can cleave the endoperoxide bridge of ARS *via* a Fe (II) Fenton-type reaction, and that free radical intermediates kill the *Plasmodia* (88, 89). Other pathways are also involved in ARTs antimalarial response (90, 91), but comprehensive research on antimalarial mechanisms remains necessary. Interestingly, as with other natural products, antimalarial properties are not the only benefits of ARTs, and ARTs have shown application value in many diseases, including cancer (**Figure 3**).

Long-term studies on the anticancer mechanism of ARTs have shown that the endoperoxide moiety is essential for its biological activity, and ARTs without the endoperoxide moiety were inactive (92, 93). In general, ARTs mainly exert anticancer effects in three ways with the endoperoxide moiety. (i) The cleavage of the endoperoxide moiety leads to the formation of ROS and oxidative stress in the tumor. Excessive production of ROS causes death by damaging cellular components, including DNA, proteins, and lipids (94); a notable feature of ARTs is that they can spontaneously generate a large amount of ROS in a heme-dependent manner (95–97). Many studies have indicated that ARTs increase the expression of cleaved caspase-3 and PARP in a variety of cancer cells by producing excessive amounts of ROS, thus inducing apoptosis in cancer cells (98–100). Further, excessive amounts of ROS may trigger an ER stress response in cancer cells (99), but the specific mechanism is not clear. Interestingly, increasing the concentration of ferrous ions and oxygen in the tumor environment to further increase the concentration of ROS has been shown to enhance the anticancer activity of ARTs (101, 102), which provides a possible strategy for the development of new anticancer drugs based on ARTs.

(ii) ARTs rely on excessive amounts of ROS to cause DNA damage in cancer cells. In the alkaline comet assay, both ARS and ART caused significant DNA damage, and the fold changes of OTM and tail DNA significantly increased (103). In addition, molecular docking indicated that various ARTs might induce DNA damage in cancer cells by inhibiting topoisomerase 1 (104), an enzyme that resolves the topological stress in genomic DNA by preventing double-stranded breaks in the DNA during cell proliferation (105). Subsequent studies involved in-depth exploration of the specific mechanism of ARTs. ATT can increase the expression of E3 ligase NEDD4, resulting in the destabilization of c-Myc protein, thereby inhibiting the expression of DNA topoisomerases. Importantly, neither a decrease in the concentration of NEDD4 protein nor DNA damage has been observed in non-cancer cells (106). DNA damage inducers such as cisplatin and doxorubicin have become the first-line cancer treatment (107, 108). However, these drugs lack strict selectivity, and are toxic to non-cancer cells. The high selectivity of ATT as a DNA damage inducer suggests that ARTs may be a potential alternative to cisplatin and doxorubicin.

(iii) ARTs induce cycle arrest to trigger cancer cell death. This anticancer effect of ARTs is closely related to excessive amounts of ROS and oxidative DNA lesions that affect cell integrity, leading to perturbations in DNA replication and cell division mechanisms. More specifically, CDK4 encodes a cyclin-dependent serine-threonine kinase in response to mitogenic or proliferation-promoting stimuli and interacts with cyclin D1 to phosphorylate the tumor suppressor protein Rb (109, 110). DHA can regulate the cyclin D1-CDK4-Rb pathway by inhibiting the expression of CDK4 to trigger cycle arrest (111). In cisplatin-resistant human breast carcinoma cells, ARS exerts anticancer activity by targeting multiple key cell cycle-related proteins, including cyclin-B1, cyclin D1, and cyclin E, to trigger cycle block (112). In general, with the help of the endoperoxide moiety, ARTs can kill cancer cells in a variety of ways. Moreover, these pathways are closely interconnected.

Ferroptosis, a new type of programmed cell death that is iron-dependent and differs from apoptosis, cell necrosis, and autophagy, has recently become a research hotspot in cancer (113–115). Interestingly, ART anticancer activity is also based on ferroptosis. Current research shows two main mechanisms of ferroptosis. In the first mechanism, the expression of the core enzyme GPX4 in the antioxidant system is reduced or inactivated, depending on the level of intracellular GSH (116). In the second mechanism, unstable iron ions accumulate in cancer cells (100). The accumulation of unstable iron ions may be the main mechanism by which ART-induced ferroptosis. The anticancer effects of ARTs are related to many iron-related proteins, including transferrin (TF), transferrin receptors 1 and 2 (TFRC, TFR2), ceruloplasmin (CP), and lactoferrin (LTF) (117). Furthermore, in DHA-induced ferroptosis, intracellular GSH levels are not affected. However, when DHA is combined with deferoxamine (DFO), an iron chelator, DHA-induced ferroptosis is completely inhibited. Further, DHA may inhibit the translation activity of a series of ferritin-related genes by maintaining the binding of iron regulatory protein-1 (IRP1) and IRP2 to iron-responsive element sequences, thus greatly increasing the concentration of unstable iron ions in cancer cells (118). However, counterintuitively, ARTs also strangely induce a negative feedback pathway in ferroptosis. ARTs can significantly increase the expression of GRP78, an antiferroptosis glucose-regulated protein, and knockdown of GRP78-enhanced ART-induced ferroptosis in AsPC-1 and PaTU8988 cells (119). DHA increases the expression of HSPA5, a negative regulator of DHA-induced ferroptosis, by activating GPX4 in glioma cells (120). It is worth considering that the induction of ferroptosis activity by ARTs could be enhanced by inhibiting the negative feedback pathway. In summary, the specific mechanism by which ARTs induce ferroptosis is still unclear, and more research is warranted. In addition, except for ferroptosis, other types of programmed cell death modes, such as apoptosis and autophagy, are also important for ARTs to kill cancer cells, and many excellent research results have been published (**Table 1**).

**Table 1:** Non- ferroptosis cancer cell death induced by ARTs.

Cell death	Cell line	Drug	Effect	Reference
apoptosis	C4, C4-2 and C4-2B prostate Ca	DHA	miR-34a↑, miR-7↑, Axl↓	Paccez et al. (121)
Apoptosis	HL-60 and KG1a leukemia	ART	cleaved caspase3↑, Bax/Bcl-2↑	Chen et al. (122)
Apoptosis	A549/TAX lung Ca	ART	Inhibit lysosome function and the clearance of dysfunctional mitochondria	Li et al. (123)
Apoptosis	4T1 and MCF-7 breast Ca	ART	HSP70↓, Bcl-2↓, cleaved caspase-9↑	Pirali et al. (124)
Autophagy-related apoptosis	RT4, RT112, T24, and TCCSup bladder Ca	ART	DNA damage↑, LC3B-I↓, LC3B-II↑	Zhao et al. (125)
Apoptosis	HCT116 and DLD-1 colorectal Ca	ART, DHA	P53↑, DR5↑, caspase-3/7↑, cleaved PARP-1↑	Zhou et al. (126)
Apoptosis	MM.1S and MM.1R multiple myeloma	ARS, DHA	ROS↑, cytochrome C translocation↑	Chen et al. (98)
Apoptosis	HT-29 and HCT-116 colorectal Ca	DHA	endoplasmic reticulum stress	Elhassanny et al. (99)
Autophagy-dependent apoptosis	T24 and EJ bladder Ca	ART	ROS↑, pAMPK↑, pmTOR↓, pULK1↑	Zhou et al. (127)
Apoptosis	EGFR-mutant and KRAS -mutant lung Ca	DHA	pSTAT3↓, Mcl-1↓, Survivin↓, Bcl-2↓	Yan et al. (128)
Apoptosis	18 types of B-cell lymphoma	ART	endoplasmic reticulum stress	Våtsveen et al. (129)
Apoptosis	CNE-2Z Nasopharyngeal Ca	DHA	CLC-3 chloride channel protein↑, cleaved caspase-3↑	Zhou et al. (130)
Autophagy	A549 lung Ca	DHA-37	HMGB1↑, pMAPK↑, LC3-II/LC3-I↑	Liu et al. (131)
Apoptosis	HCT116 colorectal Ca	ART	Inhibit the NF-κB pathway, ROS↑, Bax/Bcl-2↑	Chen et al. (132)
Apoptosis	PC3, 22RV1 and LNCaP prostate Ca	ART	Induce oxidative stress, survivin↓, cleaved PARP↑	Nunes et al. (133)

The regulation of the immune system seems to be one of the antitumor mechanisms of ARTs. This idea was first supported when ARS was directly applied to natural killer (NK) cells, and the degranulation ability of NK cells was enhanced to effectively kill cancer cells. Combination treatment with the degranulation inhibitor concanamycin A completely reversed these effects of ARS. ARS does not change the expression of activated receptors on the surface of NK cells to enhance their degranulation ability, but it could induce the activation of downstream signaling molecules (134). Subsequently, ARS can also increase the coupling between tumor and NK cells, but the expression of the main ligands of NK receptors is not affected; the specific mechanism needs to be further explored (135). Moreover, except for the innate immune system, ARTs can also regulate specific immune systems. For example, ARM can reduce Treg cells and increase IL-4 and IFN-γ in the tumor microenvironment (136). More directly, treatment with ARS in a 4T1 breast cancer model significantly reduced the number of MDSCs and Treg cells in mice and significantly increased CD4^+^ IFN-γ^+^ T cells and cytotoxic T lymphocytes (CTLs) (137). Although obvious immune regulation can be observed, few studies have investigated immune system regulation by ARTs, and the exact underlying mechanism remains unclear; however, it is still a promising research direction.

As studies on ARTs in cancer have increased, so too have the number of case reports and related clinical trials that support the potential role of ARTs in cancer treatment. There were two uveal melanoma cases in which compassionate treatment with ART was administered after ineffective standard chemotherapy (138). One patient received fotemustine plus ART and reached a temporary response, although the tumor progressed under prior fotemustine therapy alone, and the patient died 23 months after being diagnosed with stage 4 disease. The second patient reached disease stabilization after administration of dacarbazine and ART, and the survival time of the patient greatly exceeded that of the median survival time for patients with uveal melanoma, which is 2 to 5 months. Furthermore, the results of a phase I clinical study on intravenous ART in patients with advanced solid tumor malignancies were recently published (139). Nineteen patients were enrolled in the study and had various cancers. In the study, dose-limiting toxicities were observed in one of six patients at dose levels of 12 mg/kg (neutropenic fever) and 18 mg/kg (grade 3 hypersensitivity reaction on C1D1); both patients treated with 25 mg/kg experienced dose-limiting toxicities (one patient had grade 3 nausea/vomiting, and the other experienced neutropenic infection, grade 3 ALT elevation, and grade 4 ALT elevation). They also observed a disease control rate of 27% (4 out of 16). In summary, although there are issues to be resolved, ARTs have great potential as anticancer drugs.

## Progress on the Use of Quinoline Antiparasitic Drugs for Treating Cancer

**Figure 4:**
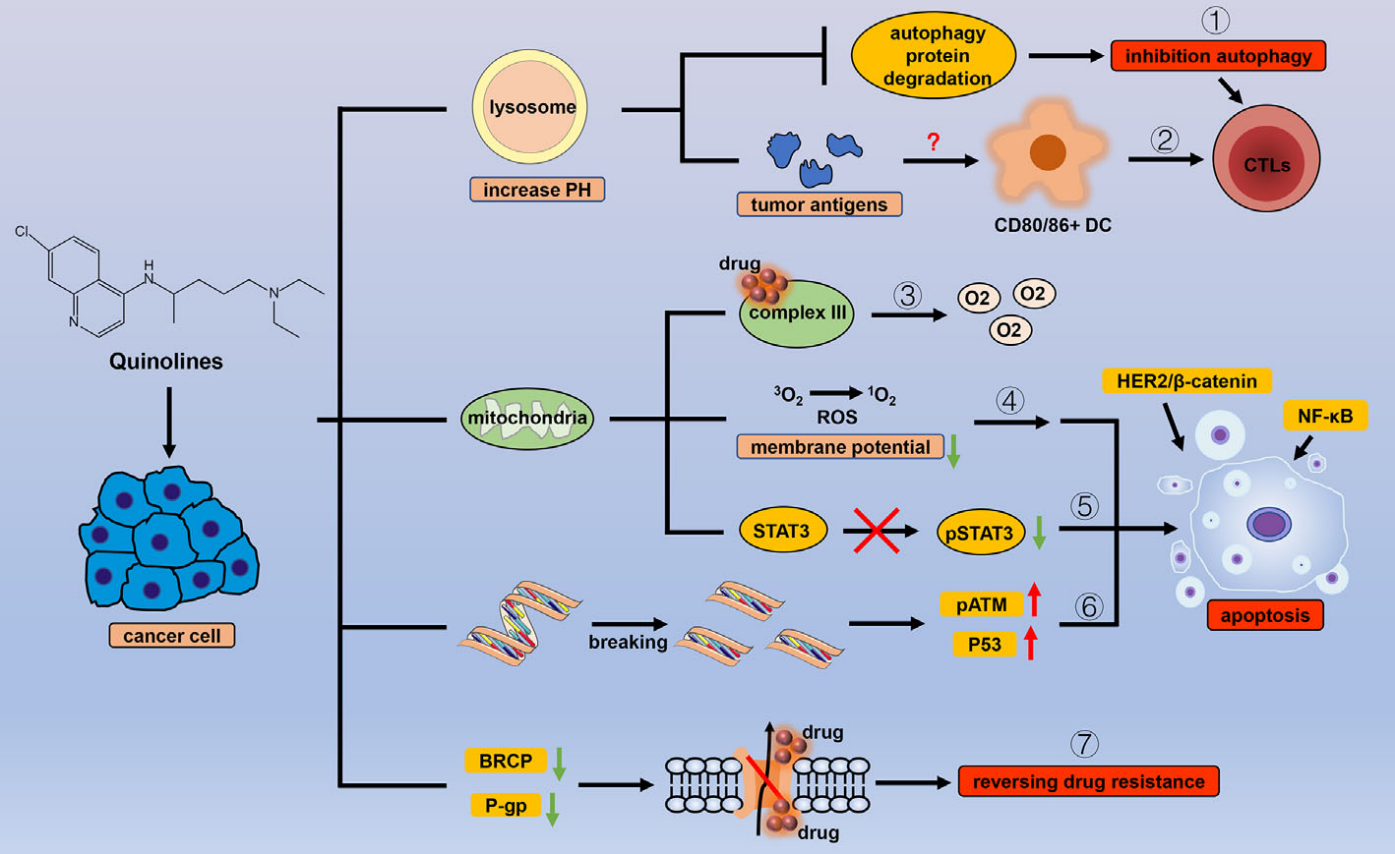
Efficacy of quinoline antiparasitic drugs in cancer. Lysosomes are an important target of quinoline drugs, and quinolines increase the pH of the lysosome, thereby triggering a variety of cascade reactions. Quinolines can (1) inhibit the degradation of autophagy proteins to block autophagy and (2) stimulate antitumor immune responses by increasing tumor antigens. Mitochondria are also a target for quinolines. Quinolines increase the oxygen concentration in cancer cells by (3) inhibiting the related processes of mitochondrial complex III and cause apoptosis by (4) inducing oxidative stress with the change in mitochondrial membrane potential, (5) inhibiting the phosphorylation level of STAT3 in mitochondria, and (6) introducing double-strand breaks in DNA in cancer cells. (7) Quinolines inhibit the drug delivery mediated by P-gp and BRCP.

Similar to ARTs, the design and synthesis of quinoline drugs, which are characterized by a quinoline ring, has been researched for application as antimalarial drugs. Quinolines exert their antimalarial effects during the blood or liver stages of the life cycle of the parasite (140), but different drugs have different mechanisms (141, 142). With the progress in research, an increasing number of quinoline drugs have shown therapeutic effects in other diseases, including cancer (**Figure 4**).

### Chloroquine

Chloroquine (CQ), a 4-aminoquinoline, has been used as an antimalarial drug for many years and is often recommended to be co-administered with primaquine to prevent recurrence of *Plasmodium vivax* (143). CQ is currently considered a protonated, weakly basic drug that increases the pH and accumulates in the food vacuole of parasites, thereby interfering with the degradation of host red blood cell hemoglobin, and preventing the growth of malaria parasite (144). The exact mechanism requires further investigation. Similar to other antiparasitic drugs, CQ has also shown potential in the treatment of cancers and other diseases (145, 146).

As lysosomotropic agents, CQ increases the pH of the lysosome from 4.5 to 6, such a change in pH is not conducive for the activity of lysosomal enzymes. This mode of action is known as a lysosomotropic effect (147, 148). Therefore, CQ can affect various biological processes by acting on receptors, enzymes, and transcriptional factors, which would determine the therapeutic effects in cancer (149) (1). Inhibit autophagy to induce anticancer effects. As an autophagy inhibitor approved by the FDA. At present, this particular function has been studied quite thoroughly. CQ blocks the final step of the autophagy process by impeding the degradation of autophagic proteins such as light chain 3B-II (LC3B-II) (150, 151). Thus, CQ prevents the production and recycling of important nutrients and metabolites, causing tumor cell damage and death. Further, the inhibition of the final stage of autophagy increases cytotoxic effects in cancer cells by promoting cell apoptosis and cell cycle arrest (152) (2). Regulate immunity to prevent tumor cells from escaping. Successive studies have shown that when ultra-low concentrations of chemotherapeutics are used on tumor cells, the expression of some genes related to inflammation, immunity, and tumor antigens in the cell increases, thereby increasing the cell’s ability to use CTLs to induce immunogenic death of tumor cells (153–157). A recent study reported for the first time the effect of low concentrations of CQ on the immunogenicity of tumor cells (158). In that study, when HCT-116 colon cancer cells were treated with CQ and 5-fluorouracil in combination, their cell lysates significantly induced the maturation of dendritic cells, and the expression of surface markers, including CD80 and CD86, was significantly increased. Additionally, dendritic cells increased the production of CTLs and triggered tumor cell death. Subsequently, they detected an increase in tumor-associated carcinoembryonic antigen family gene expression in the treated cancer cells, but the specific mechanism still needs to be explored, which may be related to the inhibition of autophagy by CQ. CQ has also been found to directly affect the function and differentiation of various immune cells by altering the pH of the lysosome and has been described in detail in this review (149).

CQ also has multiple functions in tumors by regulating a variety of key signaling molecules. Platinum drugs are recognized as the mainstay drugs for the treatment of epithelial ovarian cancer (159). However, drug-resistant cancer cells can survive the DNA damage induced by anticancer drugs through DNA repair pathways or bypassing cell cycle checkpoints (160, 161); however, CQ can upregulate the expression of p21^WAF1/CIP1^ to prevent this phenomenon and reverse the drug resistance of cancer cells. This function seems to depend on autophagy inhibition, but the connection is not yet clear (162). Many other functions of CQ depend on its inhibitory effect on autophagy. However, CQ can reduce the expression of CXC chemokine receptor 4 (CXCR4) by inhibiting STAT3 expression, thereby reducing the stemness of esophageal squamous cell carcinoma cells. This process is independent of autophagy, and the expression of key molecules, ATG7 and BECN1, in the autophagy pathway does not change (163), suggesting that CQ has other effects that do not depend on autophagy inhibition, but these have not yet been elucidated. In addition, CQ can regulate the expression of signaling molecules, including NF-κB and p53, to exert an anticancer effect (164).

As an autophagy inhibitor approved by the FDA, CQ has received widespread attention, and the possibilities of its clinical application have been extensively studied. To date, many clinical trials using CQ alone or in combination to treat cancer have been conducted (165, 166). Most findings indicate that the combination of CQ with other drugs was well tolerated, and the maximum tolerated dose increases compared with using CQ alone. However, in these trials, no significant differences between the treatment and control group and no significant improvement in overall survival were observed. This may also be related to the small sample size used in the phase I clinical trials; thus, the efficacy of CQ should be further evaluated and explored in a larger sample.

### Atovaquone

Atovaquone (ATV), a hydroxy 1,4-naphthoquinoline, is a homolog of coenzyme Q. Current research shows that ATV’s antimalarial site is the mitochondrial complex III (141). Compared with non-cancer cells, cancer cells rely more heavily on mitochondrial functions to generate ATP for growth and survival (167, 168). Naturally, respiratory energy metabolism, especially oxidative phosphorylation, has become a focus of research. ATV, an inhibitor of the mitochondrial complex III, can significantly reduce the oxygen consumption rate and increase the concentration of oxygen in cancer cells (169). In clinical practice, hypoxia is a major problem in cancer treatment (170). The current novel photodynamic therapy technology mainly relies on ROS to kill cancer cells, but the hypoxic tumor environment limits its therapeutic value (171, 172); thus, combinations with ATV may be a solution. Many studies have also revealed that ATV can indeed improve the efficacy of radiotherapy, chemotherapy, and immunotherapy by reducing the rate of oxygen consumption (173, 174).

The reduction in mitochondrial function greatly inhibits the proliferation of CSCs, which mainly rely on mitochondrial respiration rather than glycolysis. The decrease in mitochondrial membrane potential and the increase in ROS levels lead to apoptosis in CSCs; thus, ATV selectively acts on CSCs (175). More specifically, ATV can inhibit the phosphorylation of mitochondrial STAT3 but not of nuclear STAT3. The inhibition of STAT3 phosphorylation is not accompanied by changes in JAK, Src, or MEK, indicating that this function of ATV is independent of JAK/Src/MEK (176). ATV can also reduce the expression of a variety of STAT3 target genes, thereby exerting anticancer effects in tumors (177, 178). There is still much to be discovered regarding mitochondria and related functions, an aspect that has received much attention in the context of cancer treatment (179).

Mitochondrial respiratory function is not the only target of ATVs. ATVs can degrade HER2 and β-catenin in a proteasome-dependent manner, thereby inhibiting the activation of HER2/β-catenin and triggering apoptosis in cancer cells; however, application of the proteasome inhibitor MG-132 eliminates this effect of ATV (180). In addition, ATV can also introduce double-stranded breaks in DNA, thereby upregulating phosphorylated ATM and p53 to trigger cycle arrest and apoptosis in cancer cells (181). ATV is also an inhibitor of BRCP and P-gp-mediated drug delivery (182), indicating the broader application value of ATV, but its specific mechanism remains to be elucidated.

## Future Directions

People are increasingly aware of the value of drug repositioning with the urgent clinical situation that large number of new anticancer drugs have appeared unignorable drug resistance after short-term clinical use (183, 184). Encouragingly, numerous clinically approved non-cancer drugs have shown antitumor activity *in vitro*, *in vivo*, and even clinically, and antiparasitic drugs account for a large portion of these repurposed drugs (185).

Based on the current research, the application of antiparasitic drugs in anticancer therapy is multitargeted and multimodal (**Table 2**). Macrolide drugs induce cell cycle arrest, apoptosis, and autophagic death in cancer cells by regulating multiple targets and multiple signaling pathways, including WNT-TCF and PAK1-AKT-mTOR, and also play a role in reversing tumor resistance. Benzimidazole drugs induce cancer cell death by inhibiting sugar metabolism and interfering with the formation of microtubules. In particular, benzimidazoles can also inhibit the expression of HIF-1α protein to inhibit the stress behavior of cancer cells under hypoxic environments, and by changing the tumor microenvironment to stimulate the host’s antitumor immunity, benzimidazoles may also exert anticancer effects. Endoperoxide is very important for the anticancer activity of ARTs. Specifically, an endoperoxide moiety can produce a large amount of ROS in tumor cells and trigger a series of biological processes that are lethal to cancer cells. Interestingly, ARTs increase the concentration of unstable iron ions in cancer cells by regulating various ferritin and related pathways to induce ferroptosis, a new programmed cell death mode (115, 186), which may be worthwhile to study further. Similar to clinical antimalarial drugs, quinoline drugs also exert anticancer effects. CQ, a lysosomotropic agent, affects various biological pathways in cancer cells by changing the pH of lysosomes. Autophagy is currently studied more thoroughly than other mechanisms, but CQ also increases the immunogenicity of cancer cells. In addition, as a competitive inhibitor of the mitochondrial complex III, ATV can significantly affect the oxidative phosphorylation of cancer cells to change the hypoxic environment of tumor cells and greatly improve the efficacy of various clinical treatment methods. At present, the application of antiparasitic drugs in tumor treatment is not only at the stage of theoretical experimentation, but several clinical trials have already been carried out to analyzed the feasibility of specific drugs.

**Table 2:** Antitumor mechanism of antiparasitic drugs.

Category	Drug	Drug action	Effect
Macrolides	Avermectins	Apoptosis	Inhibit Cl^-^Channel, Mitochondrial related pathways
		Cell cycle	Cycle-related protein, KPNB1-dependent
		Autophagy	Ubiquitination pathway, PAK1↓, Inactivate AKT-mTOR
		Cell stemness	Inhibit stemness genes, Inactivate PAK1, pStat3↓, IL-6↓
	Milbemycins	Drug resistance	MDR1↓, P-gp↓, MAST1↓
Benzimidazoles	Albendazole	Energy homeostasis	Inhibition GLUT1/AMPK/P53, HIF-1α↓
		Cell cycle	Inhibit microtubule formation
		Apoptosis	ER stress, pMAPK↑, TNF-α↑
	Flubendazole	Cell cycle	Inhibiting tubulin polymerization
		Autophagy	EVA1A↑, LC3 puncta↓, p62 degradation↓, and LC3 lipidation↓, STAT3 related pathways
		Immunity	PD-1↓, accumulation of MDSCs↓
Artemisinin and derivatives	Artemisinin	DNA damage	ROS↑, topoisomerase 1↓
		Cell cycle	Cycle-related protein (cyclin-B1, cyclin D1, cyclin E)
		Immunity	Degranulation of NK cells↑, MDSCs and Treg cells↓, CD4 +IFN-γ+ T and CTL↑
	Artesunate	DNA damage	ROS↑
	Artemisitene	DNA damage	NEDD4↑, the stability of c-Myc↓, DNA topoisomerases↓
	Dihydroartemisinin	Cell cycle	ROS↑, CDK4↓
		Ferroptosis	Iron-related proteins, Binding of iron IRP1 and IRP2↑, unstable iron ions↑
	Artemether	Immunity	Treg↓, IL-4 and IFN-γ↑
Quinolines	Chloroquine	Autophagy	Impeding the degradation of autophagic proteins
		Immunity	tumor-associated carcinoembryonic antigen↑, CD80 and CD86↑
		Drug resistance	p21WAF1/CIP1↑
		Cell stemness	STAT3↓, CXCR4↓
	Atovaquone	Energy homeostasis	Inhibiting mitochondrial complex III, Oxidative phosphorylation↓
		Cell stemness	mitochondrial membrane potential↓, ROS↑, pSTAT3 in mitochondrial↓
		Apoptosis	Degrade HER2 and β-catenin, pATM and p53↑

In summary, antiparasitic drugs are involved in almost all aspects of tumors, including cell cycle, apoptosis, autophagy, ferroptosis, stress, energy homeostasis, immunity, and drug resistance. Besides, when used in combination with existing clinical tumor drugs, many antiparasitic drugs show significant synergistic effects (187–189). However, according to current research results, antiparasitic drugs are still far from being repurposed into antitumor drugs that can be widely used in clinical practice. The current research on the anticancer mechanisms of antiparasitic drugs is still not comprehensive enough, and more thorough research is needed. In addition, antiparasitic and tumor therapy have two different application environments, and many problems remain to be solved. The first problem is drug delivery; the external microenvironment of the parasite-infected foci is relatively normal, whereas the growth of tumors mainly depends on glycolysis, which leads to an acidic external microenvironment. Whether this microenvironment affects the delivery of drugs needs further exploration. The second problem is drug concentration. Different drug concentrations cause different dominant effects. The concentration that has the best anticancer effect and does not cause side effects in the human body needs to be established. The third problem is that although antiparasitic drugs have many advantages, we cannot rule out they might promote tumor growth. Studies have shown that CQ-induced stress in cancer cells can activate NF-κB, thereby conferring transcriptional and phenotypic plasticity to cells, resulting in the reprogramming of cells and allows tumor cells to escape cell death induced by either drug therapy or the immune system (190, 191). Fortunately, for the first two issues, many studies have conducted in-depth study. Increasing evidence shows that nanotechnology-based drug delivery methods yield better therapeutic effects at lower concentrations and might be clinically implemented in the near future (192–198). Successive preclinical studies and clinical trials have clarified in detail the therapeutic effects of different drug concentrations and various possible side effects. However, there are very few studies have investigated the cancer-promoting effects of antiparasitic drugs. However, this may be very important for us to fully understand the role of antiparasitic drugs in tumors. After understanding these cancer-promoting effects, with the help of modern drug modification and improvement technologies (199–202), it can greatly accelerate the real application of antiparasitic drugs in clinical cancer treatment. More problems are likely to be encountered as research and practice progress. Nevertheless, it is undeniable that antiparasitic drugs indeed have great potential for development as broad-spectrum, clinically applicable antitumor drugs.

## Author Contributions

J-GD and HZ contributed substantially to the design of this review and gave the approval of the final version for publishing. DZ and JZ prepared the table. Q-XL and XL prepared the figures. Y-QL and ZZ wrote the manuscript. All authors contributed to the article and approved the submitted version.

## Funding

This work was supported by grants from the National Natural Science Foundation of China (No. 81972190), Natural Science Foundation of Chongqing (cstc2018jcyjAX0178) and the Scientific Research Project of Undergraduate, the Army (Third Military) Medical University (2019XBK48).

## Conflict of Interest

The authors declare that the research was conducted in the absence of any commercial or financial relationships that could be construed as a potential conflict of interest.
